# Supplementary material for: Coastal lake sediments from Arctic Svalbard suggest colder summers are stormier
Source: Nat Commun. 2024 Nov 11;15:9688. doi: 10.1038/s41467-024-53875-1 (PMC11555044; doi:10.1038/s41467-024-53875-1)
Supplement: Supplementary file 1 — Supplementary Information [file 41467_2024_53875_MOESM1_ESM.pdf]

Supplementary Information

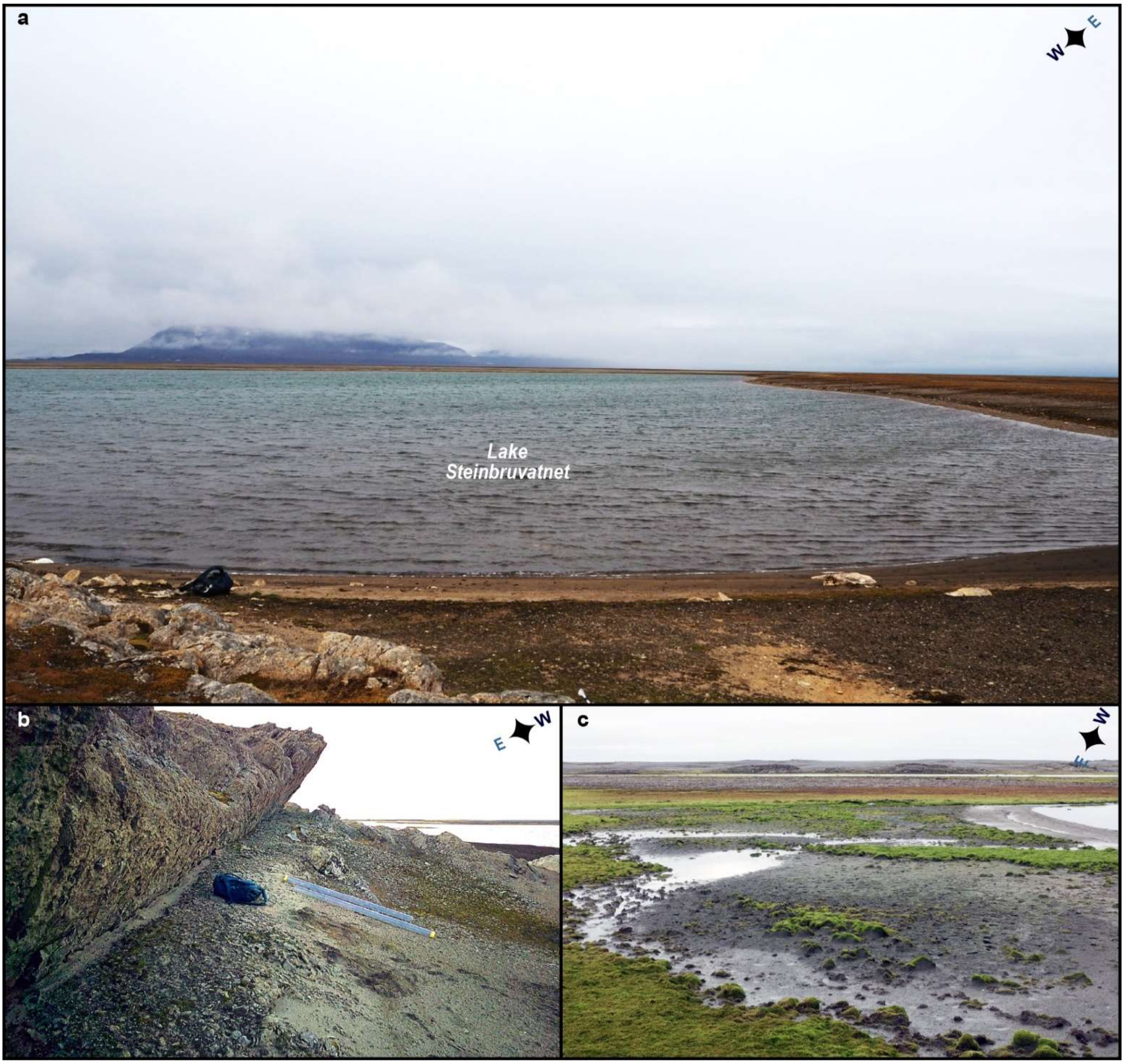

Figure S1. Catchment geomorphology. a View over Lake Steinbruvatnet from the southwest (photo by M. C. Strzelecki). **b** The base of the rocky ridge that shelters Lake Steinbruvatnet from the westerlies with the shadow dune sampled for CS 1 (see setting and Fig. S4 – photo by C. J. Hein). **c** Silty sheet sampled for CS 2 (see Fig. S4) to the East of Lake Steinbruvatnet. Also, note the numerous cushion-like tundra plants growing in beach ridge swales and shaped like miniature parabolic dunes (photo by S. Lindhorst).

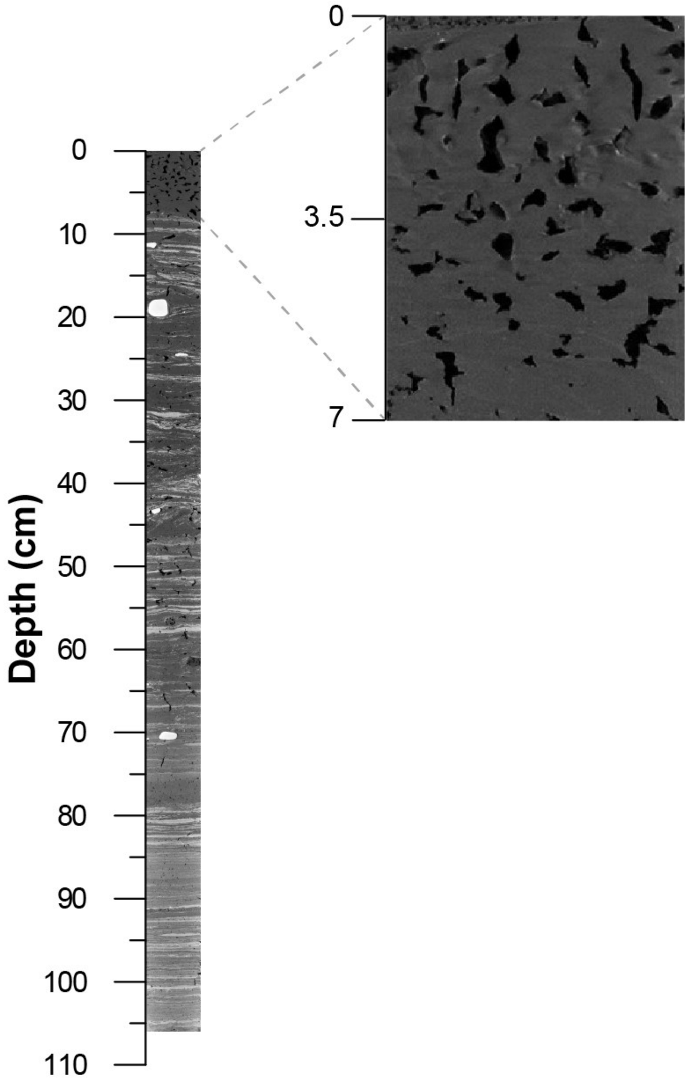

Figure S2. CT orthoslice of analyzed core 601-21-6-GC with a close-up of the top 7 cm. Visual assessment reveals a porous and homogenized composition, which is markedly different from the underlying 99 cm (see results and discussion section in the main text).

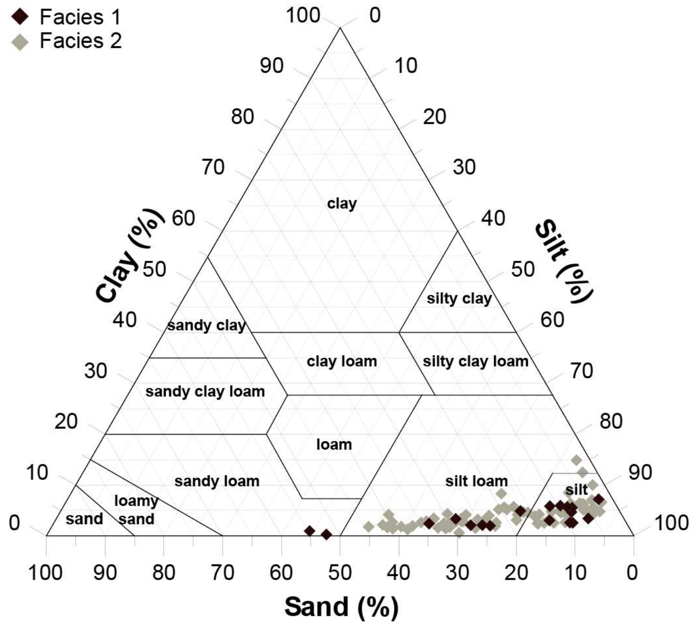

Figure S3. Soil texture classification diagram. GRADISTAT-derived (see methods section) abundances (%) of sand, silt, and clay in all (*n*=97) grain size samples from working core 601-21-6-GC[1]. Facies 1-2 are shown in colors that match those of Fig. 3 in the main text.

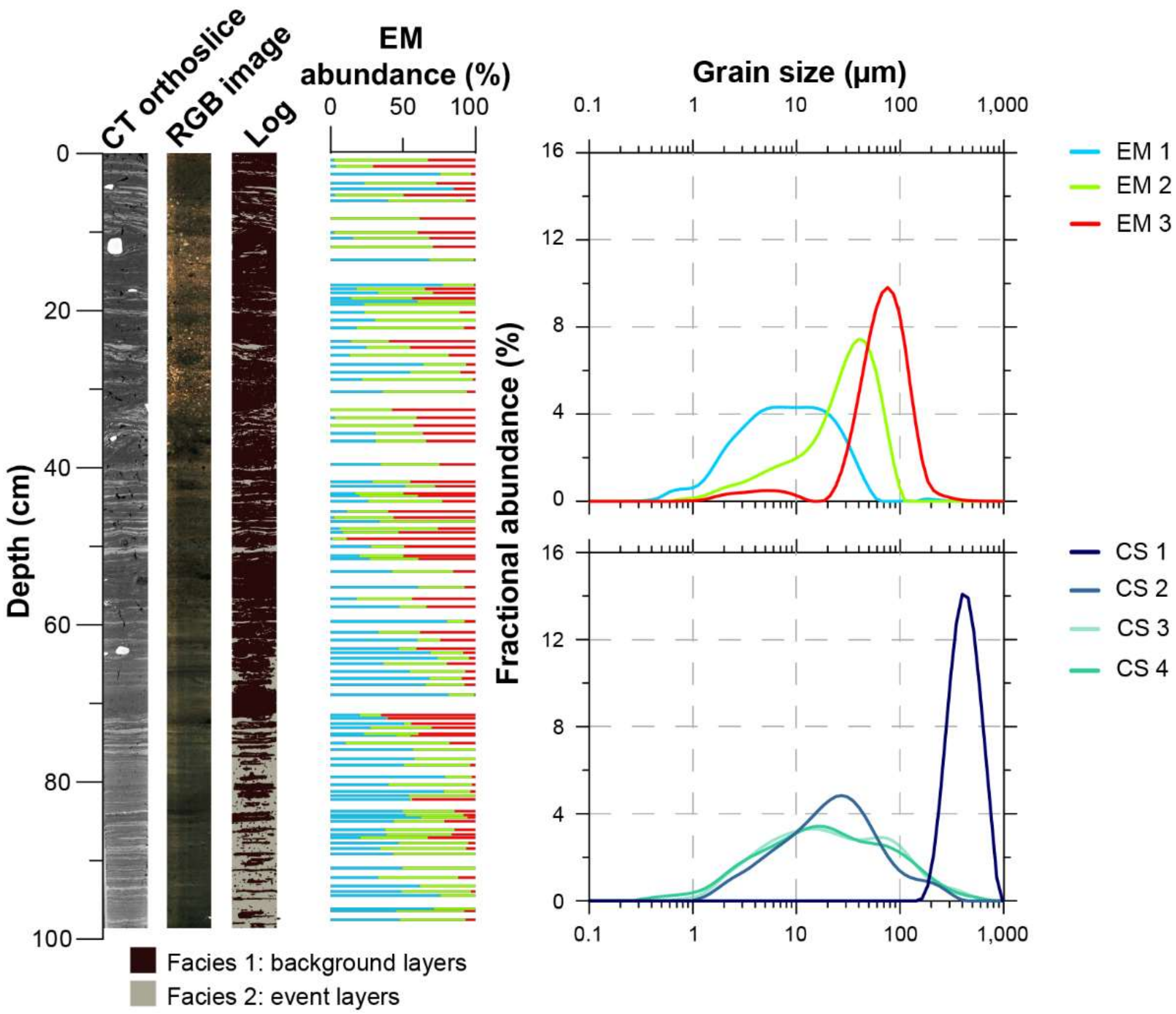

Figure S4. End-Member Modelling Analysis (EMMA) results. From left to right: CT and RGB imagery of investigated core 601-21-6 GC, a log with facies 1 and 2 highlighted, down-core variability in EM abundance (%), the particle size distributions (PSDs) of each EM (top right) as well as catchment samples (CS; bottom right) 1-4 from the western (1), and eastern (2) shores, respectively, as well as from the eolian silt sheets from the south-east transect from Lake Steinbruvatnet towards the modern beach (see the Holocene evolution of Steinbruvatnet and Fig. 1d in the main text).

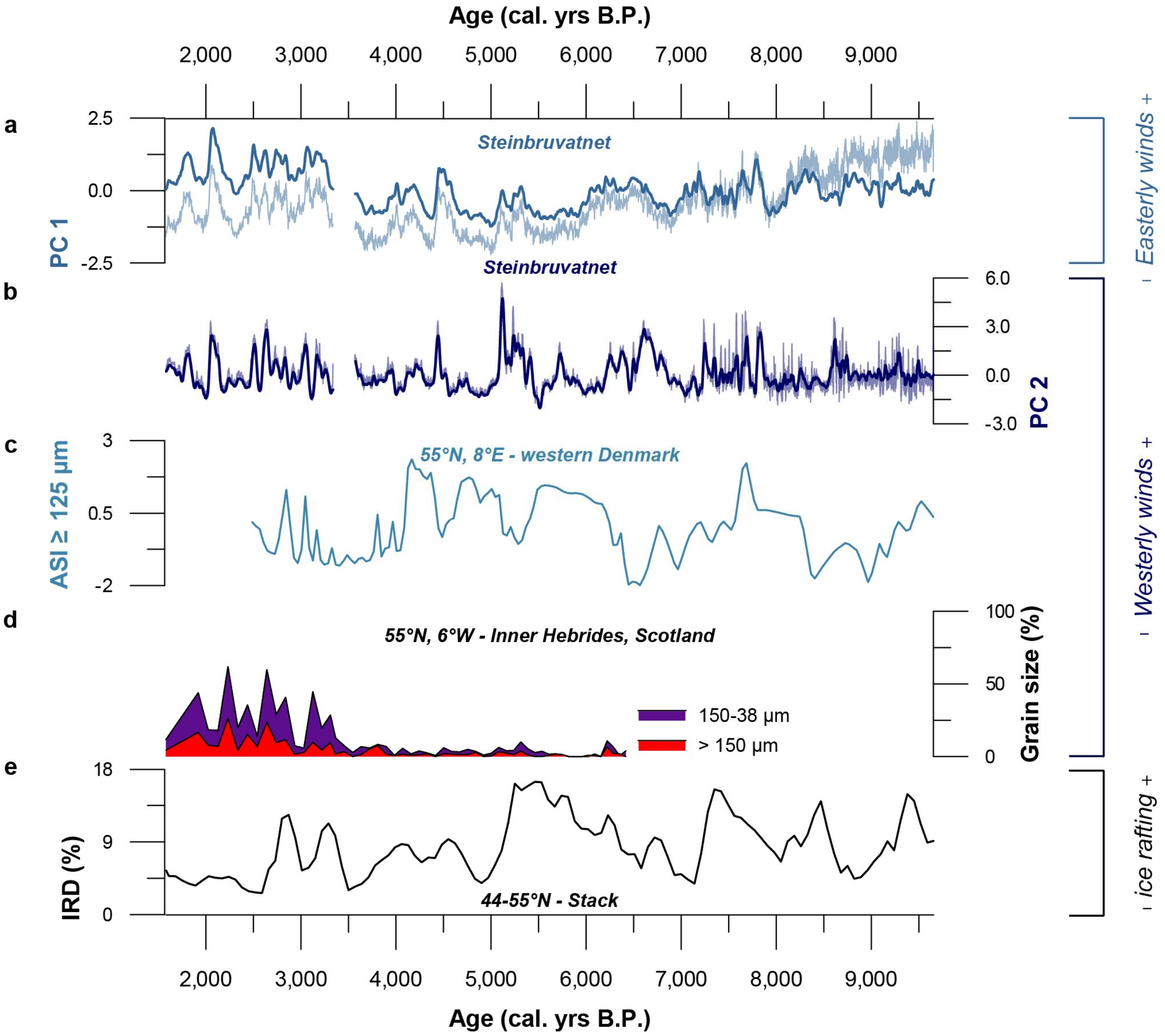

Figure S5. Holocene changes in wind regime further contextualized. A comparison between our PC-based wind reconstructions and relevant reconstructions of storminess and paleoclimate data not shown in Fig. 5 of the main text. Localities are indicated in Fig. 1a using matching colors. **a-b** Easterly and westerly storm tracks captured by PC 1-2 values, respectively. 30-year moving averages are shown in bold, while the raw data has not been detrended as in Fig. 5 of the main manuscript. **c** Aeolian Sand Influx (ASI) to Lake Filsø in western Denmark by [3]. **d** grain-size variability from the Laphroaig coastal peat bog in southwestern Scotland by [4], and the Ice Rafted Debris (IRD) stack from the North Atlantic by [5,6].

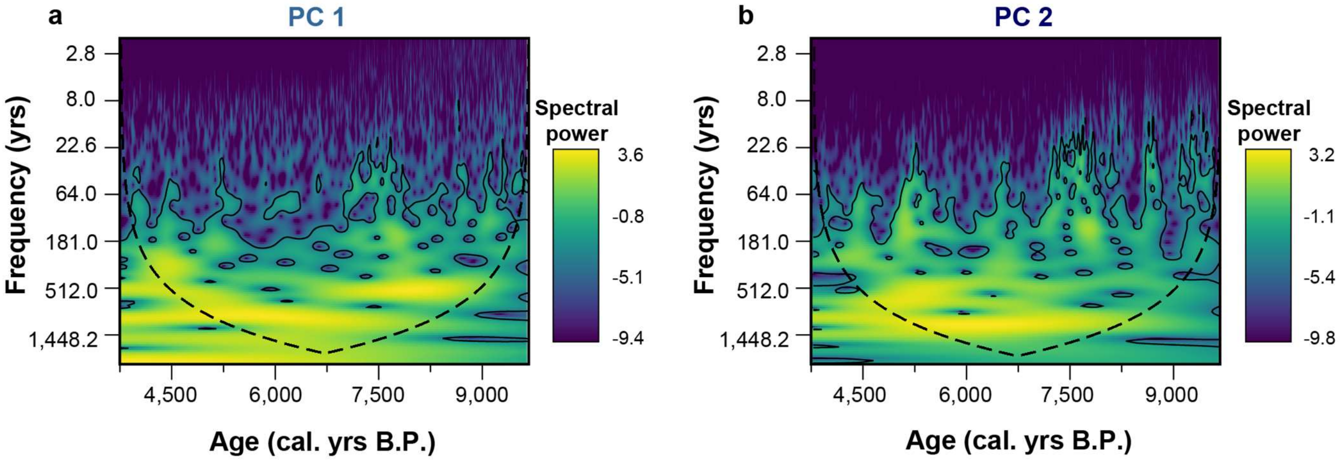

Figure S6. Continuous wavelet transform (CWT) analysis. The scalograms highlight the prevalence of a ~1,500 cyclicity in our: **a** PC 1 (polar Easterlies) and **b** PC 2 (Westerlies) data. The dashed black line marks the cone of influence – signals outside this area might be distorted[7]. The 95% significance interval is highlighted using the black contour.

Supplementary Note 1: Geomorphological controls of storm impacts and eolian transport

Storm waves that approach Sørkappøya are effectively prevented from penetrating inland by the rocky skerries, -ridges, and -submarine platform that surround the island and act as wave breakers (see Fig. 1d). In addition, the lake itself is protected from the west by at least two rocky ridges, rising to 12 m and 7 m above modern sea level (see Fig. 1d). All of this paints a picture of a system that – despite its location in one of the most storm-exposed locations in the Svalbard Archipelago – is resistant to direct storm impacts like surges.

In keeping with the above interpretation, we did not trace overwash features or wave-induced erosion of uplifted ridge surfaces during fieldwork. The absence of overwash features or storm surge deposits in the modern coastal environment confirms the effectiveness of the structural geomorphology in protecting the island from storm surges[8]. In line with this evidence, the preservation of periglacial features on the surface of the uplifted beach ridge plain, and the development of a network of ice-wedge polygons on the marine terraces (north shore of the

lake), indicate the presence of permafrost. Infiltration or inundation by saline water would not have allowed the formation and subsequent preservation of these features[9–11].

As also outlined in the main text, we argue that cryogenic factors such as the presence of sea ice, ice foot, snow cover and lake ice are important factors limiting sediment transport and deposition, as in most Arctic eolian environments[12–14]. Apart from ice and snow, permafrost also strongly influences processes on Svalbard – frozen ground conditions and seasonal freezing in our study area effectively bind surface sediments to the surface for most of the year (October-June), limiting sediment availability for transport to the thaw period[15].

References:

1. Blott, S. J. & Pye, K. GRADISTAT: a grain size distribution and statistics package for the analysis of unconsolidated sediments. *Earth Surf. Process. Landforms* **26**, 1237–1248 (2001).
2. Prins, M. A. & Weltje, G. J. End-member modeling of siliciclastic grain-size distributions: The late Quaternary record of aeolian and fluvial sediment supply to the Arabian Sea and its paleoclimatic significance. in *Numerical experiments in stratigraphy: Recent advances in stratigraphic and sedimentologic computer simulations* (ed. Harbaugh, J.) 91–111 (Society for Sedimentary Geology, 1999).
3. Goslin, J. *et al.* Holocene centennial to millennial shifts in North-Atlantic storminess and ocean dynamics. *Sci Rep* **8**, 12778 (2018).
4. Kylander, M. E. *et al.* It's in your glass: a history of sea level and storminess from the Laphroaig bog, Islay (southwestern Scotland). *Boreas* **49**, 152–167 (2020).
5. Bond, G. *et al.* Persistent solar influence on North Atlantic climate during the Holocene. *Science* **294**, 2130–2136 (2001).

6. Evans, M. N. *et al.* NOAA/WDS Paleoclimatology - Bond et al. 2001 North Atlantic Holocene Drift Ice Proxy Data. NOAA National Centers for Environmental Information https://doi.org/10.25921/BGVH-NB23.
7. Torrence, C. & Compo, G. P. A Practical Guide to Wavelet Analysis. *Bull. Amer. Meteor. Soc.* **79**, 61–78 (1998).
8. Trenhaile, A. Rocky coasts — their role as depositional environments. *Earth-Science Reviews* **159**, 1–13 (2016).
9. Nitzbon, J. *et al.* Pathways of ice-wedge degradation in polygonal tundra under different hydrological conditions. *The Cryosphere* **13**, 1089–1123 (2019).
10. Strzelecki, M. C. *et al.* Cryo-conditioned rocky coast systems: A case study from Wilczekodden, Svalbard. *Science of The Total Environment* **607–608**, 443–453 (2017).
11. Murton, J. B. & Kolstrup, E. Ice-wedge casts as indicators of palaeotemperatures: precise proxy or wishful thinking? *Progress in Physical Geography: Earth and Environment* **27**, 155–170 (2003).
12. Forbes, D. L. & Taylor, R. B. Ice in the shore zone and the geomorphology of cold coasts. *Progress in Physical Geography* **18**, 59–89 (1994).
13. Byrne, M.-L. & Dionne, J.-C. Typical Aspects of Cold Regions Shorelines. in *Landscapes of Transition: Landform Assemblages and Transformations in Cold Regions* (eds. Hewitt, K., Byrne, M.-L., English, M. & Young, G.) 141–158 (Springer Netherlands, Dordrecht, 2002). doi:10.1007/978-94-017-2037-3_7.
14. Seppälä, M. Wind as a Geomorphic Agent in Cold Climates. *Cambridge University Press* (2004).
15. Rymer, K. G. *et al.* Contemporary and past aeolian deposition rates in periglacial conditions (Ebba Valley, central Spitsbergen). *CATENA* **211**, 105974 (2022).
